# Development and Validation of a Novel Indicator for Differential Diagnosis of Clinically Significant Prostate Cancer Based on Comprehensive Hematological Testing

**DOI:** 10.3390/diagnostics16121884

**Published:** 2026-06-17

**Authors:** Fangming Wang, Yuzhe Tang, Gang Zhang, Jianxing Li

**Affiliations:** Department of Urology, Beijing Tsinghua Changgung Hospital, School of Clinical Medicine, Tsinghua Medicine, Tsinghua University, No. 168, Litang Road, Changping District, Beijing 102218, China; tyza00873@btch.edu.cn (Y.T.); zga02851@btch.edu.cn (G.Z.)

**Keywords:** clinically significant prostate cancer, hematological indicators, coagulation parameters, PSA density, discrimination index, PSA gray zone

## Abstract

**Objective:** This study aims to explore new hematological indicators with differential diagnostic significance for clinically significant prostate cancer (csPCa) by conducting comprehensive hematological tests, and to construct a novel discrimination index (DI) (csPCa-DI) to improve the diagnostic accuracy of csPCa. **Methods:** A total of 542 patients suspected of prostate cancer who were admitted to Beijing Tsinghua Changgung Hospital from November 2014 to May 2025 were enrolled in this study. All patients underwent complete blood count, coagulation testing, full biochemical analysis, and prostate biopsy. According to the biopsy results, patients were divided into the csPCa group and the non-csPCa group. The differences in hematological indicators between the two groups were compared, and multivariate logistic regression analysis was used to screen independent risk factors for csPCa. Two scoring systems (Fib-PLT Score and Fib-PLT-DD Score) were constructed based on coagulation-related parameters, and a csPCa discrimination index (csPCa-DI) was further established by integrating independent risk factors. The diagnostic efficacy of these scores, csPCa-DI, and traditional PSA-related indicators was evaluated by receiver operating characteristic (ROC) curves. Stratified validation was performed in the PSA gray zone (4–10 ng/mL) population. **Results:** Multivariate logistic regression identified prostate-specific antigen density (PSAD) (OR = 18.063, 95% CI: 7.125–45.792, *p* < 0.001), age (OR = 1.062, 95% CI: 1.024–1.102, *p* = 0.001), and Fib-PLT-DD Score (OR = 0.388, 95% CI: 0.251–0.599, *p* < 0.001) as independent predictors of csPCa. Based on the regression coefficient (β) weights of the independent predictors, the csPCa-DI was formulated as: csPCa-DI = 2.894 × PSAD + 0.060 × Age − 0.946 × Fib-PLT-DD Score (Fib-PLT-DD Score = 0.672 × Fib + 0.008 × PLT − 0.028 × DD). In the overall cohort, the area under the ROC curve (AUC) of csPCa-DI for diagnosing csPCa was 0.821 (95% CI: 0.773–0.868, *p* < 0.001), higher than that of total PSA (0.701) and f/t PSA ratio (0.727), and slightly higher than PSAD (0.797). The optimal cut-off value of csPCa-DI in the overall cohort was 1.46 points, with a sensitivity of 87.4% and specificity of 61.8%. In the PSA gray zone population, csPCa-DI exhibited superior diagnostic efficacy with an AUC of 0.736 (95% CI: 0.634–0.838, *p* < 0.001), significantly higher than total PSA (0.465) and f/t PSA ratio (0.638), and slightly higher than PSAD (0.720). A csPCa-DI cut-off value of 0.92 points in the PSA gray zone achieved a high sensitivity of 96.0% (specificity = 48.0%), effectively reducing missed diagnosis, while the cut-off of 1.46 points balanced sensitivity (68.0%) and specificity (70.7%). **Conclusions:** The novel csPCa-DI constructed by integrating PSAD, age, and coagulation-derived Fib-PLT-DD Score based on β weights has higher diagnostic efficacy for csPCa than traditional single PSA-related parameters, especially in the PSA gray zone, which can provide a new clinical tool for the screening and differential diagnosis of csPCa. This study also clarifies the correlation between local coagulation abnormalities and csPCa, providing a new perspective for understanding the pathological mechanism of csPCa.

## 1. Introduction

Prostate cancer (PCa) ranks among the most common malignancies in elderly men and represents the second leading cause of cancer-related mortality worldwide in the male population [1]. Clinically significant PCa (csPCa) was defined as Gleason score ≥ 7 or International Society of Urological Pathology (ISUP) Grade Group ≥ 2 (https://pubmed.ncbi.nlm.nih.gov/26492179/) (accessed on 6 June 2026) [2], which necessitates active therapeutic intervention due to its inherent potential for progression and metastasis, which is distinct from non-clinically significant PCa (non-csPCa), which can be managed with active surveillance [3]. Early and accurate discrimination of csPCa is crucial for improving patient prognosis, reducing unnecessary medical interventions, and alleviating the socio-economic burden caused by overdiagnosis and overtreatment [4]. In routine clinical practice, multiparametric magnetic resonance imaging (mpMRI) is usually combined with conventional PSA-related indicators to jointly stratify PCa risk and guide subsequent biopsy decisions [5].

At present, total prostate-specific antigen (tPSA) and free/total PSA ratio (f/t PSA) are the most commonly used clinical screening indicators for csPCa [6,7,8]. However, their diagnostic efficacy has obvious limitations, especially within the tPSA gray zone (4–10 ng/mL), where biomarker levels overlap substantially between benign and malignant prostate lesions. For example, data on gray-zone PCa incidence indicate that only approximately 25% of men are ultimately diagnosed with PCa, of whom roughly half harbor clinically significant disease. Consequently, over 80% of patients undergo unnecessary invasive biopsy procedures [9,10]. Therefore, developing a more accurate and reliable diagnostic tool based on routine clinical indicators has become an urgent clinical need.

Recently, emerging biomarkers such as the Prostate Health Index and 4K score panel, as well as novel liquid biopsy signatures, have been proposed to improve the diagnostic performance for csPCa [11]. However, these advanced tests are costly and poorly accessible in primary care settings, limiting their large-scale population screening. Moreover, studies have found that inflammatory factors and coagulation indicators are closely related to the occurrence and development of csPCa [12,13,14,15]. However, most prior studies only focused on one or a few individual hematological markers, failing to systematically and comprehensively explore the correlation between a full spectrum of routine blood parameters and csPCa. In view of this research gap, this study aims to establish a novel marker for the early identification of clinically significant prostate cancer in patients scheduled for prostate biopsy to assist clinicians in formulating biopsy strategies for localized prostate cancer. We conducted a comprehensive screening of routine hematological indicators potentially associated with csPCa, covering coagulation profiles, blood routine and biochemical parameters, and further constructed a coagulation-integrated novel discrimination index (DI) for csPCa (csPCa-DI), and then verified its diagnostic value, aiming to provide a practical auxiliary tool to complement the current mpMRI- and PSA-based diagnostic workflow for csPCa identification and screening, especially for patients in the PSA gray zone with low diagnostic accuracy of traditional indicators.

## 2. Materials and Methods

### 2.1. Study Population

Patients suspected of PCa at Beijing Tsinghua Changgung Hospital between November 2014 and May 2025 were included in this study. The inclusion criteria were: (1) patients aged ≥50 years; (2) patients who underwent initial transrectal ultrasound-guided prostate biopsy; (3) patients who completed comprehensive hematological tests (complete blood count, coagulation profile, full biochemical panel) within 1 week before biopsy. The exclusion criteria were: (1) biopsy results being unclear or incomplete; (2) incomplete clinical data or hematological test results; (3) concurrent acute infection, autoimmune diseases, or other diseases that may affect hematological indicators; (4) history of other malignant tumors, severe liver and kidney dysfunction, coagulation disorders, or anticoagulant treatment; (5) previous surgical treatment history for benign prostatic hyperplasia (BPH); (6) patients diagnosed with metastatic PCa; (7) previously diagnosed, recurrent or advanced PCa. Finally, 542 eligible patients were included in the study. This retrospective study was approved by the Ethics Committee of Beijing Tsinghua Changgung Hospital. All procedures were performed in accordance with the principles of the Declaration of Helsinki. Since all data were anonymized, informed consent was waived.

### 2.2. Grouping Criteria

All patients underwent transrectal ultrasound-guided prostate biopsy, combining a standard 12-core systematic scheme with 1–2 targeted cores obtained from suspicious lesions identified on MRI and real-time ultrasound, and the biopsy specimens were pathologically examined by two senior pathologists independently. According to the pathological results, patients were divided into two groups: the csPCa group and the non-csPCa group. csPCa was defined as PCa with a Gleason score ≥ 7 points (ISUP ≥ 2); non-csPCa included BPH, prostatitis, and PCa with a Gleason score < 7 (ISUP < 2).

### 2.3. Hematological Testing and Data Collection

Venous blood samples were taken at admission after 8 h fasting and before any treatment. The blood samples were sent to the Department of Laboratory Medicine, Beijing Tsinghua Changgung Hospital, for testing within 2 h. Complete blood count indicators [white blood cells (WBCs), red blood cells (RBCs), hemoglobin (HGB), hematocrit (HCT), mean corpuscular volume (MCV), mean corpuscular hemoglobin (MCH), mean corpuscular hemoglobin concentration (MCHC), red cell distribution width—standard deviation (RDW-SD), red cell distribution width—coefficient of variation (RDW-CV), platelet (PLT), plateletcrit (PCT), platelet distribution width (PDW), mean platelet volume (MPV), platelet–large cell ratio (P-LCR), neutrophil count (NEU#), lymphocyte count (LY#), monocyte count (MONO#), eosinophil count (EOS#), basophil count (BASO#), neutrophil percentage (NEUT%), lymphocyte percentage (LY%), monocyte percentage (MONO%), eosinophil percentage (EOS%), basophil percentage (BASO%)] were detected by an automatic hematology analyzer (Sysmex XN-9000, Kobe, Japan). Coagulation indicators [(thrombin time (TT), prothrombin time (PT), prothrombin time percentage (PT%), prothrombin time ratio (PTR), international normalized ratio (INR), activated partial thromboplastin time (APTT), fibrinogen (Fib), D-dimer, fibrin degradation product (FDP)] were detected by an automatic coagulation analyzer (Stago STA-R, Asnières-sur-Seine, France). Full biochemical panel indicators [alanine transaminase (ALT), aspartate transaminase (AST), alkaline phosphatase (ALP), gamma-glutamyl transferase (GGT), total bilirubin (TBIL), direct bilirubin (DBIL), total protein (TP), albumin (ALB), pre-albumin (Pre-ALB), total bile acid (TBA), glucose (GLU), blood urea nitrogen (BUN), creatinine (Cre), uric acid (UA), calcium (Ca), inorganic phosphorus (IP), potassium (K), sodium (Na), chloride (Cl), total cholesterol (TC), triglyceride (TG), high-density lipoprotein cholesterol (HDL-C), low-density lipoprotein cholesterol (LDL-C), small dense low-density lipoprotein cholesterol (sd LDL-C), creatine kinase (CK), lactate dehydrogenase (LDH)] were detected by an automatic biochemical analyzer (Roche Cobas 8000, Mannheim, Switzerland). PSA-related indicators (tPSA, fPSA) were detected by chemiluminescence immunoassay (Beckman Coulter DXI 800, Brea, CA, USA), and PSAD was calculated as tPSA divided by prostate volume (prostate volume was measured on MRI using the ellipsoid formula: transverse diameter × anteroposterior diameter × craniocaudal diameter × 0.52). All testing procedures were performed in accordance with the operating instructions of the instruments, and quality control was carried out to ensure the accuracy of the test results.

### 2.4. Construction of Scoring Systems and csPCa-DI

Based on the results of multivariate logistic regression analysis, the β coefficients of the independent risk factors were used to construct the scoring systems and csPCa-DI. The Fib-PLT Score was constructed using the β coefficients of Fib and PLT (Fib-PLT Score = 0.672 × Fib + 0.008 × PLT). The Fib-PLT-DD Score was constructed by adding the β coefficient of D-dimer to the Fib-PLT Score (Fib-PLT-DD Score = 0.672 × Fib + 0.008 × PLT − 0.028 × D-dimer). The csPCa-DI was constructed by integrating the β coefficients of PSAD, age, and Fib-PLT-DD Score (csPCa-DI = 2.894 × PSAD + 0.060 × Age − 0.946 × Fib-PLT-DD Score).

### 2.5. Statistical Analysis

All statistical analyses were performed using SPSS 22.0 software (SPSS Inc., Chicago, IL, USA) and GraphPad Prism 8.0 statistical software (GraphPad Software Inc., La Jolla, CA, USA). Measurement data were tested for normality by the Shapiro–Wilk test. Continuous variables with normal distribution were expressed as mean ± standard deviation and compared using independent-sample *t*-test; non-normally distributed variables were expressed as median (interquartile range) and compared using Mann–Whitney U test. Categorical variables were expressed as frequency (percentage) and compared using the χ^2^ test. Multivariate logistic regression analysis was applied to evaluate the prediction efficacy of PSA-derived parameters, each coagulation-related indicator and the novel constructed csPCa-DI for predicting PCa in four different models. The diagnostic efficacy of csPCa-DI and other indicators were evaluated by receiver operating characteristic (ROC) curves, and the area under the curve (AUC), 95% confidence interval (95% CI), sensitivity, specificity, and Youden index were calculated. The null hypothesis was that the true AUC = 0.5. *p* < 0.05 was considered statistically significant. The DeLong test was used to statistically compare the AUC values between csPCa-DI and PSAD. Net reclassification improvement (NRI) and integrated discrimination improvement (IDI) were calculated to quantify the incremental predictive ability of csPCa-DI relative to PSAD. NRI > 0 and IDI > 0 with *p* < 0.05 representing significant predictive improvement. Internal validation of the csPCa-DI model was performed using 1000 bootstrap resampling replicates to evaluate model robustness and stability. The Hosmer–Lemeshow goodness-of-fit test was used to assess model calibration, whereby a *p*-value > 0.05 indicated satisfactory consistency between predicted and observed probabilities.

## 3. Results

### 3.1. Baseline Characteristics of the Two Groups

**Figure 1:**
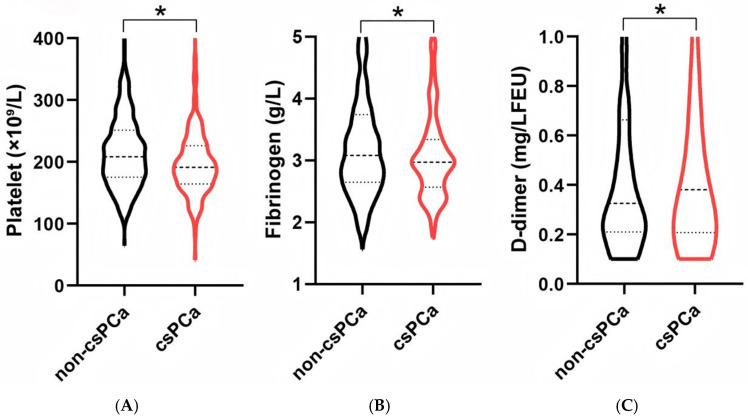
Platelet, fibrinogen, and D-dimer levels in non-csPCa and csPCa patients. Violin plots display (**A**) platelet count, (**B**) fibrinogen, and (**C**) D-dimer distributions in non-csPCa (black) and csPCa (red) groups. Dashed lines denote median and quartiles. * *p* < 0.05. Abbreviations: csPCa, clinically significant prostate cancer; non-csPCa, non-clinically significant prostate cancer. Dashed lines denote median and quartiles.

A total of 542 patients were enrolled in this study, including 355 cases in the non-csPCa group and 187 cases in the csPCa group. The baseline characteristics of the two groups are shown in Table 1. There were significant differences in age, WBCs, RBCs, HGB, HCT, MCV, MCH, RDW-SD, PLT, PCT, PDW, EOS%, Fib, D-dimer, tPSA, fPSA, f/t PSA ratio, and PSAD between the two groups (all *p* < 0.05). The csPCa group had higher age, RDW-SD, PDW, EOS%, D-dimer, tPSA, fPSA, and PSAD, and lower WBCs, RBCs, HGB, HCT, MCV, MCH, PLT, PCT, and f/t PSA ratio compared with the non-csPCa group. There were no significant differences in other indicators (BMI, MCHC, RDW-CV, MPV, P-LCR, NEU#, LY#, MONO#, EOS#, BASO#, NEUT%, LY%, MONO%, BASO%, TT, PT, PT%, PTR, INR, APTT, FDP, ALT, AST, ALP, GGT, TBIL, DBIL, TP, ALB, Pre-ALB, TBA, GLU, BUN, Cre, UA, Ca, IP, K, Na, Cl, TC, TG, HDL-C, LDL-C, sd LDL-C, CK, LDH) between the two groups (all *p* > 0.05). The violin plots of PLT, Fib, and D-dimer levels in the two groups are shown in Figure 1, which clearly shows the distribution differences of these three coagulation indicators between the non-csPCa and csPCa groups (all *p* < 0.05).

### 3.2. Multivariate Logistic Regression Analysis of csPCa Risk Factors

To screen independent risk factors for csPCa, multivariate logistic regression analysis was performed with the presence of csPCa as the dependent variable and the indicators with significant differences as independent variables. We adopted a multi-step strategy for variable selection. (1) Univariate analysis was used to screen all demographic, hematological, inflammatory and coagulation indicators; variables with *p* < 0.05 were selected as candidate variables. (2) Combined with existing clinical evidence, we reserved markers proven to be associated with the occurrence and development of prostate cancer, and removed indicators with limited clinical application value. (3) Multicollinearity diagnosis was carried out, and variables with obvious collinearity were excluded to guarantee model reliability. (4) Forward stepwise logistic regression was applied to further filter independent predictors. Specifically, all 65 enrolled indicators were subjected to intergroup baseline comparison (Table 1), and 18 variables with *p* < 0.05 were screened out for subsequent regression analysis. Next, univariate logistic regression was performed based on the above 18 parameters; seven hematological indices including RBCs, HGB, HCT, MCV, MCH, RDW-SD and EOS% showed *p* > 0.05 and were eliminated. The remaining 11 variables were further assessed for multicollinearity via variance inflation factor (VIF, cut-off = 5). Five indicators with VIF > 5 were removed: PCT and PDW (highly collinear with PLT), tPSA, fPSA and f/t PSA ratio (highly collinear with PSAD). Ultimately, six variables (age, WBCs, PLT, Fib, D-dimer, PSAD) were incorporated into forward stepwise multivariate logistic regression to screen independent predictive factors. We further added BMI and ALB into the multivariate regression models as essential clinical covariates as BMI and ALB represent patients’ metabolic and nutritional–inflammatory status, which are common confounding factors in PCa predictive models and showed no multicollinearity with other variables. Finally, we selected age, WBCs, ALB, and the three coagulation-related parameters [PLT, Fib, D-dimer] for subsequent regression analysis to explore their predictive value for csPCa. The reason was as follows: age is a well-recognized independent risk factor for PCa and a critical covariate that must be adjusted in clinical prediction models; WBCs reflect systemic inflammation, which is closely linked to tumor microenvironment remodeling and cancer progression; ALB is a marker of nutritional status and systemic inflammation; and PLT, Fib, and D-dimer are core components of the coagulation cascade, and emerging evidence demonstrates their close association with tumor angiogenesis, invasion, and metastatic potential in prostate cancer [16,17,18].

Two regression models were established (Table 2). Model 1 included age, BMI, WBCs, ALB, Fib, PLT, and PSAD. The results showed that age (β = 0.061, OR = 1.063, 95% CI: 1.025–1.102, *p* = 0.001), Fib (β = −0.672, OR = 0.511, 95% CI: 0.346–0.754, *p* = 0.001), PLT (β = −0.008, OR = 0.992, 95% CI: 0.986–0.997, *p* = 0.005), and PSAD (β = 2.939, OR = 18.892, 95% CI: 7.417–48.117, *p* < 0.001) were independent risk factors for csPCa. Model 2 included age, BMI, WBCs, ALB, D-dimer, PLT, and PSAD. The results showed that age (β = 0.057, OR = 1.059, 95% CI: 1.020–1.098, *p* = 0.002), PLT (β = −0.009, OR = 0.992, 95% CI: 0.986–0.997, *p* = 0.003), and PSAD (β = 2.638, OR = 13.989, 95% CI: 5.716–34.235, *p* < 0.001) remained independent risk factors for csPCa, while D-dimer was not an independent risk factor (*p* = 0.801).

### 3.3. Construction and Diagnostic Efficacy of Coagulation-Related Scoring Systems

**Figure 2:**
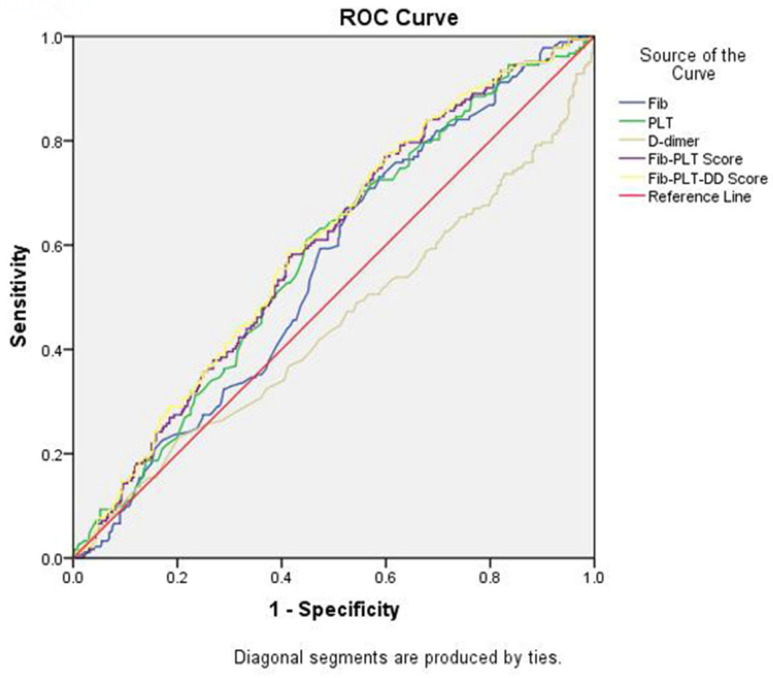
Receiver operating characteristic (ROC) curves of coagulation-related parameters for the diagnosis of csPCa in all enrolled subjects. Abbreviations: csPCa, clinically significant prostate cancer; Fib, fibrinogen; PLT, platelet count.

Based on the β coefficients of Fib, PLT, and D-dimer in multivariate regression analysis, two coagulation-related scoring systems (Fib-PLT Score and Fib-PLT-DD Score) were constructed. The diagnostic efficacy of these two scoring systems for csPCa is shown in Table 3 and Table 4. Multivariate regression analysis including the scoring systems showed that Fib-PLT Score (β = −0.956, OR = 0.384, 95% CI: 0.250–0.590, *p* < 0.001) and Fib-PLT-DD Score (β = −0.946, OR = 0.388, 95% CI: 0.251–0.599, *p* < 0.001) were independent risk factors for csPCa when combined with age, BMI, WBCs, ALB, and PSAD. ROC curve analysis showed that the AUC of Fib-PLT Score was 0.595 (95% CI: 0.546–0.645, *p* < 0.001), and the AUC of Fib-PLT-DD Score was 0.602 (95% CI: 0.553–0.652, *p* < 0.001), indicating that both scoring systems had certain diagnostic efficacy for csPCa. The ROC curves of the coagulation-related parameters for csPCa diagnosis are shown in Figure 2.

### 3.4. Diagnostic Efficacy of csPCa-DI and PSA-Related Indicators

**Figure 3:**
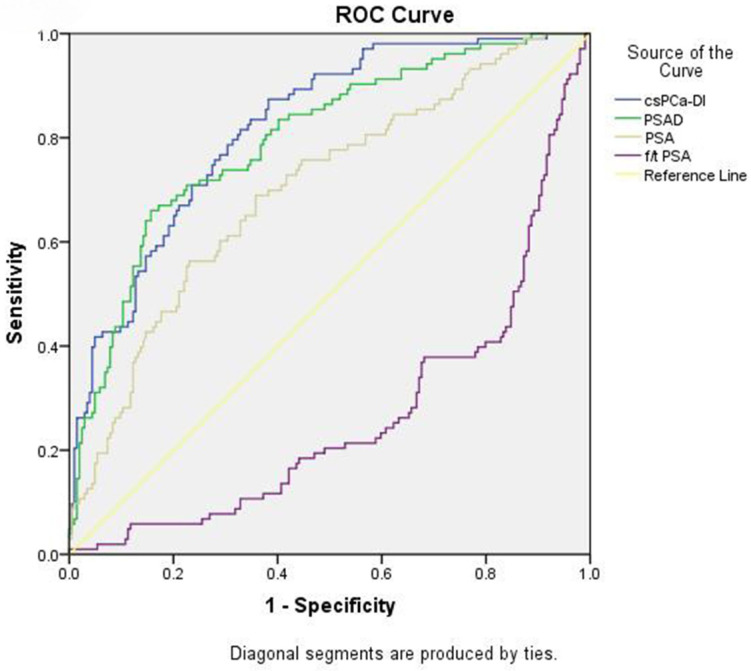
Receiver operating characteristic (ROC) curves of csPCa-DI and PSA-related parameters for the diagnosis of csPCa in all enrolled subjects. Abbreviations: csPCa, clinically significant prostate cancer; csPCa-DI: csPCa discrimination index; PSAD, prostate-specific antigen density; f/t PSA, free/total prostate-specific antigen ratio.

The diagnostic efficacy of csPCa-DI and PSA-related indicators in the total enrolled population is shown in Table 5. ROC curve analysis showed that csPCa-DI had the highest AUC (0.821, 95% CI: 0.773–0.868, *p* < 0.001) among all predictors, followed by PSAD (AUC = 0.797, 95% CI: 0.744–0.850, *p* < 0.001), f/t PSA ratio (AUC = 0.727, 95% CI: 0.680–0.774, *p* < 0.001), and tPSA (AUC = 0.701, 95% CI: 0.639–0.763, *p* < 0.001). The optimal cut-off value of csPCa-DI was 1.46, with a sensitivity of 87.4%, a specificity of 61.8%, and a Youden index of 0.492. The ROC curves of csPCa-DI and PSA-related indicators in the total population are shown in Figure 3. Bootstrap internal validation showed that the regression coefficient of csPCa-DI remained stable, with a 95% bootstrap confidence interval of 0.690–1.382 (*p* = 0.001). The Hosmer–Lemeshow test confirmed good model calibration (χ^2^ = 7.269, df = 8, *p* = 0.508), suggesting no obvious deviation between predicted risk and actual csPCa incidence. The AUC of csPCa-DI was 0.821, while the AUC of single PSAD was 0.797. DeLong’s test verified a statistically significant difference between the two AUC values (*p* = 0.032). Further calculation showed NRI = 0.186 (95% CI: 0.092–0.280, *p* < 0.001) and IDI = 0.058 (95% CI: 0.021–0.095, *p* = 0.002), suggesting that csPCa-DI significantly improved classification and discrimination performance compared with isolated PSAD.

### 3.5. Validation of Diagnostic Efficacy in the PSA Gray Zone

Of all 542 participants, 204 patients had PSA values in the 4–10 ng/mL gray zone, including 38 csPCa cases. To validate the diagnostic performance of the newly constructed csPCa-DI in the clinically challenging PSA gray zone (4–10 ng/mL), we further analyzed the subgroup of patients with tPSA levels in this range. The validation results of diagnostic efficacy are presented in Table 6. ROC curve analysis demonstrated that csPCa-DI still maintained good diagnostic validity in this subgroup (AUC = 0.736, 95% CI: 0.634–0.838, *p* < 0.001), confirming its applicability in the population with ambiguous tPSA results. For clinical practicality, we further determined two cut-off values of csPCa-DI: when the cut-off value was set to 0.92, the diagnostic sensitivity reached 96.0% with a specificity of 48.0%, which is suitable for preliminary screening of csPCa patients in the PSA gray zone to reduce missed diagnoses; when the cut-off value was adjusted to 1.46, the specificity increased to 70.7% with a sensitivity of 68.0%, which can be used for further confirmation of csPCa to avoid unnecessary invasive biopsies.

**Figure 4:**
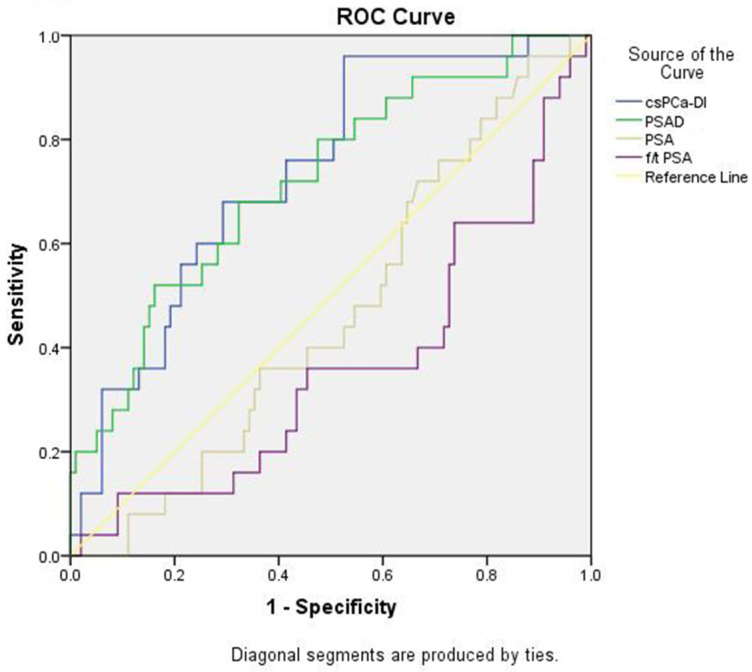
Receiver operating characteristic (ROC) curves of csPCa-DI and PSA-related parameters for the diagnosis of csPCa in subjects with gray-zone PSA. Abbreviations: csPCa, clinically significant prostate cancer; csPCa-DI: csPCa discrimination index; PSAD, prostate-specific antigen density; f/t PSA, free/total prostate-specific antigen ratio. The reference line represents the non-discriminatory test (AUC = 0.5).

In contrast, the validation results of traditional PSA-related indicators showed that PSAD had a relatively good diagnostic efficacy (AUC = 0.720, 95% CI: 0.608–0.832, *p* = 0.001) with a cut-off value of 0.18, corresponding to a sensitivity of 52.0% and a specificity of 83.8%. The f/t PSA ratio exhibited moderate diagnostic validity (AUC = 0.638, 95% CI: 0.512–0.763, *p* = 0.034), with an optimal cut-off value of 0.175, a sensitivity of 60.0%, and a specificity of 71.7%. Notably, tPSA, the most commonly used clinical screening indicator, failed to show significant diagnostic value in the PSA gray zone (AUC = 0.465, 95% CI: 0.349–0.581, *p* = 0.586), and no valid cut-off value could be obtained. These validation results are consistent with the core objective of this study, confirming that the comprehensive hematological indicator-based csPCa-DI has superior diagnostic performance compared with traditional PSA-related indicators in the PSA gray zone, and can effectively make up for the limitations of tPSA in this range. The ROC curves of csPCa-DI and PSA-related indicators in the PSA gray zone are shown in Figure 4.

## 4. Discussion

This study comprehensively performed complete blood count, coagulation profile, and full biochemical panel tests to explore new hematological indicators with differential diagnostic significance for csPCa, and constructed coagulation-related scoring systems and a novel csPCa-DI. The results showed that comprehensive hematological testing can effectively identify potential indicators related to csPCa, and the constructed csPCa-DI has good diagnostic efficacy for csPCa, especially in the PSA gray zone, which provides a new clinical tool for csPCa screening and discrimination without expensive equipment or invasive procedures.

The major innovation of this study lies in the comprehensive and systematic screening strategy of hematological indicators. Previous relevant studies mostly focused on one or two isolated markers, lacking an overall analysis of multi-dimensional routine blood parameters, which led to the failure of fully mining the potential diagnostic value of conventional laboratory indicators [14,15,16,17,18].

Consistent with existing evidence, tumor-related coagulation and inflammatory activation are closely associated with the malignant progression of PCa [16,17]. Nevertheless, our study differs obviously from those studies in both study design and key findings. Both previous studies focused on PCa patients without setting non-csPCa subjects as a control group. Yu Z et al. found that PLT, Fib, and D-dimer levels in the bone metastasis group were evidently higher than those in the non-bone metastasis group in the PCa cohort [16]. Lei X et al. reported that Fib and D-dimer exhibit significant positive correlation with high-risk PCa [17]. In contrast, we enrolled non-csPCa patients as controls and observed lower Fib levels in the csPCa group, which was inconsistent with two previous studies. Pathologically, PCa activates local coagulation through tissue factor to establish a hypercoagulable state. Fib and PLT are largely consumed within the tumor microenvironment, resulting in their decreased concentrations in peripheral blood [18]. Extensive microthrombosis further induces secondary fibrinolysis and increases D-dimer [19]. This coagulation–fibrinolysis imbalance indicates disrupted tumor microenvironment and corresponds to the biological behavior of csPCa. We should also interpret the reduced Fib and PLT levels in the csPCa group with caution. In general, elevated levels of these two markers correlate with aggressive disease and poor prognosis in PCa. The unexpected inverse correlation may stem from selection bias, varied disease stages or unadjusted confounders. The underlying mechanism needs further basic research to be verified. Multivariate logistic regression analysis confirmed that higher age and PSAD were independent risk factors for csPCa, while lower Fib and PLT count were independently associated with an increased risk of csPCa after adjusting for confounding factors, D-dimer showed no independent predictive value in the adjusted model. Based on the regression coefficients of independent risk factors for csPCa, we constructed two coagulation-related scoring systems (Fib-PLT Score and Fib-PLT-DD Score). The results showed that both scoring systems remained independently and significantly correlated with csPCa and had certain diagnostic efficacy for csPCa, with AUCs of 0.595 and 0.602, respectively. The addition of D-dimer to the Fib-PLT Score did not significantly improve the diagnostic efficacy, which may be because D-dimer is not an independent risk factor for csPCa in this study. This is inconsistent with some previous studies [12,13], which may be related to the differences in the study population and sample size. The csPCa-DI constructed by integrating PSAD, age, and Fib-PLT-DD Score showed the best diagnostic efficacy among all predictors, with an AUC of 0.821 in the total population, which was superior to that of traditional PSA-related indicators. Notably, although the absolute difference in AUC between csPCa-DI and PSAD seems moderate, DeLong’s test, NRI and IDI consistently confirmed the statistical superiority of csPCa-DI.

Knowledge regarding the performance of PSA derivatives for detecting PCa and csPCa remains limited in men with PSA levels within the gray zone. Several studies showed that the differences in tPSA level and f/t PSA between PCa and non-PCa group were not significant among patients with tPSA in the gray zone [20,21,22,23]. Liu J et al. reported that PSAD was independent clinical parameter for predicting csPCa in the PSA gray-zone population, with an AUC of 0.68 [22]. In our study, in the PSA gray-zone population, csPCa-DI still exhibited the highest diagnostic performance for csPCa with an AUC of 0.736 (*p* < 0.001), which was superior to PSAD (AUC = 0.720), f/t PSA (AUC = 0.638), and tPSA (AUC = 0.465; *p* = 0.586, no significant efficacy). This indicates that csPCa-DI can effectively improve the diagnostic accuracy of csPCa, especially for patients in the PSA gray zone, which can help reduce unnecessary prostate biopsies. The optimal cut-off value of csPCa-DI was 1.46, with a sensitivity of 87.4% and a specificity of 61.8%, which balances the sensitivity and specificity, making it suitable for clinical screening. In addition, when the cut-off value was adjusted to 0.92, the sensitivity reached 96.0%, which can be used for early screening of high-risk populations; when the cut-off value was adjusted to 1.46, the specificity reached 70.7%, which can be used for further confirmation of csPCa.

Compared with conventional PSA-related diagnostic indicators, the newly developed csPCa-DI exhibits favorable cost-effectiveness for clinical application. All variables included in csPCa-DI are derived from routine hematological, biochemical, and coagulation tests that are routinely performed in clinical practice. No additional venous blood collection, specialized testing kits, or high-cost molecular assays are required. Therefore, csPCa-DI achieves improved diagnostic accuracy, especially in the PSA gray zone, without bringing extra economic burden to patients, which makes it more cost-effective and suitable for large-scale population screening and primary clinical promotion than traditional single PSA detection. Clinically, the csPCa-DI can be applied at both pre-MRI and post-MRI stages of prostate cancer diagnosis. Before mpMRI examination, it can be used for preliminary risk stratification in patients with elevated PSA to screen high-risk individuals who require further imaging evaluation. After mpMRI, csPCa-DI can be combined with the prostate imaging reporting and data system (PI-RADS) scores to refine biopsy decision-making, thereby optimizing the diagnostic workflow and reducing unnecessary invasive procedures.

In line with contemporary clinical practice, the updated 2026 guideline jointly released by the American Urological Association (AUA) and Society of Urologic Oncology (SUO) emphasizes multimodal risk stratification for early prostate cancer detection. The guideline strongly recommends integrating novel biomarkers, liquid biopsy techniques, and artificial intelligence-based diagnostic models to optimize risk assessment, improve the detection of clinically significant prostate cancer, and reduce excessive and unnecessary prostate biopsies [5]. In this context, our newly developed csPCa-DI, derived from routine hematological and coagulation parameters, conforms to the latest diagnostic trends of minimally invasive, cost-effective, and multimodal risk stratification, providing a feasible supplementary tool for current clinical screening systems.

This study has some limitations. First, this is a single-center retrospective study, and the sample size is limited. As a retrospective analysis, data collection was not prospectively designed for this research topic, which may introduce potential selection bias and weaken the representativeness and external generalization of the results. In addition, although we conducted bootstrap internal validation and Hosmer–Lemeshow calibration analysis to verify model performance, this study did not perform head-to-head comparison with existing published prostate cancer nomograms. Further multicenter prospective studies with larger sample sizes are needed to compare the diagnostic efficiency of csPCa-DI with classic predictive models and external cohorts. Second, the study did not explore the mechanism of the association between hematological indicators and csPCa, which needs to be further studied in the future. Third, we acknowledge that incorporating additional diagnostic instruments and clinical indicators, including mpMRI and genomic markers, could have further enhanced our novel csPCa-DI, and these factors may be integrated in future studies. Fourth, the csPCa-DI is based on hematological tests, so prostatitis and inflammatory conditions may cause false positives. Subgroup analysis for these patients was not feasible in this retrospective study. Prospective studies are needed to clarify such interference and verify the performance of this index. Fifth, this study primarily assessed the discriminative ability of the included parameters using conventional diagnostic metrics. No clinical risk thresholds for biopsy were predefined, so decision curve analysis (DCA) was not carried out. Further studies targeting clinical decision-making are needed to explore the net clinical benefit of these biomarkers with DCA. Sixth, csPCa was defined only by biopsy Gleason score in this study. Prostate biopsy sampling bias and tumor heterogeneity may cause pathological undergrading.

## 5. Conclusions

Comprehensive hematological testing covering complete blood counts, coagulation profiles, and full biochemical panels may help identify potential hematological indicators related to csPCa. Age, Fib, PLT, and PSAD are independent risk factors for csPCa. The constructed Fib-PLT Score, Fib-PLT-DD Score, and csPCa-DI have good diagnostic efficacy for csPCa, and csPCa-DI shows the best performance, especially in the PSA gray zone. The newly developed csPCa-DI may serve as a simple, non-invasive supplementary indicator for the screening and differential diagnosis of csPCa, and help facilitate early diagnosis and optimize patient prognosis. However, as a novel marker established based only on a single-center retrospective cohort without external validation, its generalizability remains limited. Further multicenter prospective studies are warranted to verify its diagnostic performance and clinical applicability before large-scale clinical application.

## Figures and Tables

**Table 1:** Baseline characteristics of the two groups.

Indicators	Non-csPCa (*n* = 355)	csPCa (*n* = 187)	*p* Value
Age (years)	69.97 ± 8.59	74.63 ± 8.47	**<0.0001**
BMI (kg/m^2^)	24.92 ± 3.43	24.57 ± 3.46	0.2735
WBCs (×10^9^/L)	6.45 ± 2.21	6.08 ± 1.67	**0.042**
RBCs (×10^12^/L)	4.48 ± 0.56	4.30 ± 0.57	**0.0003**
HGB (g/L)	136.60 ± 16.89	132.50 ± 17.16	**0.0078**
HCT (%)	40.04 ± 4.66	38.82 ± 4.82	**0.0043**
MCV (fL)	89.49 ± 4.58	90.46 ± 4.05	**0.0151**
MCH (pg/Cell)	30.51 ± 1.85	30.86 ± 1.73	**0.0325**
MCHC (g/L)	340.90 ± 9.80	341.10 ± 9.12	0.7486
RDW-SD (fL)	41.80 (39.80–43.80)	42.80 (40.70–44.40)	**0.0019**
RDW-CV (%)	12.70 (12.20–13.20)	12.80 (12.40–13.30)	0.1069
PLT (×10^9^/L)	208.00 (175.00–251.00)	191.00 (164.00–226.00)	**0.0005**
PCT (%)	0.22 ± 0.05	0.20 ± 0.05	**0.0011**
PDW (fL)	12.30 (10.68–15.45)	13.00 (11.20–16.00)	**0.0047**
MPV (fL)	10.24 ± 1.00	10.30 ± 1.09	0.4672
P-LCR (%)	26.88 ± 7.67	27.60 ± 7.92	0.3001
NEU# (×10^9^/L)	3.59 (2.81–4.41)	3.41 (2.76–4.29)	0.0944
LY# (×10^9^/L)	1.62 (1.28–2.00)	1.63 (1.35–2.03)	0.6326
MONO# (×10^9^/L)	0.48 (0.39–0.60)	0.48 (0.40–0.58)	0.895
EOS# (×10^9^/L)	0.14 (0.08–0.23)	0.16 (0.10–0.26)	0.075
BASO #(×10^9^/L)	0.03 (0.02–0.04)	0.02 (0.02–0.04)	0.8263
NEUT (%)	59.90 (53.60–65.80)	58.60 (53.30–64.80)	0.0719
LY (%)	27.80 (22.60–33.40)	29.20 (24.20–34.40)	0.1694
MONO (%)	7.90 (6.70–9.40)	8.00 (7.10–9.50)	0.2488
EOS (%)	2.40 (1.50–3.90)	3.00 (1.70–4.20)	**0.0339**
BASO (%)	0.40 (0.30–0.60)	0.40 (0.30–0.60)	0.8264
TT (s)	17.42 ± 2.81	17.42 ± 0.83	0.9741
PT (s)	11.83 ± 0.76	11.88 ± 0.76	0.4585
PT% (%)	97.83 ± 11.44	96.61 ± 11.31	0.2340
PTR	1.01 ± 0.07	1.02 ± 0.07	0.3799
INR	1.01 ± 0.07	1.02 ± 0.07	0.2043
APTT (s)	27.63 ± 2.19	27.67 ± 2.21	0.8277
Fib (g/L)	3.08 (2.65–3.74)	2.97 (2.57–3.34)	**0.0168**
D-dimer (mg/LFEU)	0.33 (0.21–0.66)	0.38 (0.21–1.04)	**0.0465**
FDP (mg/L)	2.50 (2.50–2.50)	2.50 (2.50–2.75)	0.0500
ALT (U/L)	15.45 (11.10–22.83)	15.20 (11.30–20.60)	0.4764
AST (U/L)	17.45 (13.88–20.73)	17.80 (15.15–21.25)	0.0918
ALP (U/L)	67.00 (58.00–82.00)	71.00 (58.00–86.88)	0.1431
GGT (U/L)	21.00 (16.00–31.23)	19.00 (14.45–29.00)	0.0652
TBIL (μmol/L)	10.80 (7.98–15.16)	11.45 (8.52–14.70)	0.7319
DBIL (μmol/L)	4.40 (3.45–5.71)	4.77 (3.80–5.90)	0.1236
TP (g/L)	66.19 ± 5.53	65.66 ± 5.22	0.2834
ALB (g/L)	41.01 ± 3.72	40.71 ± 3.36	0.3681
Pre-ALB (g/L)	0.25 (0.21–157.50)	0.24 (0.20–151.70)	0.2350
TBA (μmol/L)	5.00 (3.00–6.20)	5.00 (3.10–7.53)	0.5853
GLU (mmol/L)	5.83 ± 1.61	5.77 ± 1.60	0.6778
BUN (mmol/L)	6.52 ± 3.44	6.64 ± 3.42	0.6871
Cre (μmol/L)	80.00 (72.00–92.00)	80.00 (72.93–96.00)	0.7131
UA (μmol/L)	337.00 (287.00–401.00)	351.00 (287.50–409.00)	0.4607
Ca (mmol/L)	2.27 ± 0.11	2.27 ± 0.11	0.9328
IP (mmol/L)	1.09 ± 0.18	1.07 ± 0.16	0.4314
K (mmol/L)	4.05 ± 0.39	4.08 ± 0.35	0.2677
Na (mmol/L)	141.50 ± 2.40	141.30 ± 2.27	0.3804
Cl (mmol/L)	105.60 ± 2.96	105.50 ± 2.75	0.8206
TC (mmol/L)	4.26 ± 0.92	4.22 ± 0.94	0.6422
TG (mmol/L)	1.14 (0.84–1.59)	1.12 (0.89–1.51)	0.8607
HDL-C (mmol/L)	1.12 ± 0.31	1.13 ± 0.27	0.9270
LDL-C (mmol/L)	2.61 ± 0.83	2.54 ± 0.85	0.4128
sd LDL-C(mmol/L)	0.60 (0.48–0.85)	0.61 (0.43–0.91)	0.5442
CK (U/L)	75.50 (53.23–104.80)	79.00 (60.53–102.20)	0.5387
LDH (U/L)	168.30 ± 32.56	172.7 ± 43.76	0.1866
tPSA (ng/mL)	9.50 (6.14–15.54)	20.87 (10.60–42.18)	**<0.0001**
fPSA (ng/mL)	1.86 (1.22–3.12)	2.37 (1.40–5.80)	**0.0001**
f/t PSA ratio	0.20 (0.15–0.30)	0.12 (0.09–0.18)	**<0.0001**
PSAD (ng/mL^2^)	0.15 (0.10–0.27)	0.48 (0.25–1.05)	**<0.0001**

Note: Continuous variables with normal distribution were expressed as mean ± standard deviation and compared using independent-sample *t*-test; non-normally distributed variables were expressed as median (interquartile range) and compared using Mann–Whitney U test. *p* < 0.05 was considered statistically significant and the bold values indicate statistical significance. Abbreviations: csPCa, clinically significant prostate cancer; BMI, body mass index; WBCs, white blood cells; RBCs, red blood cells; HGB, hemoglobin; HCT, hematocrit; MCV, mean corpuscular volume; MCH, mean corpuscular hemoglobin; MCHC, mean corpuscular hemoglobin concentration; RDW-SD, red blood cell distribution width—standard deviation; RDW-CV, red blood cell distribution width—coefficient of variation; PLT, platelet; PCT, plateletcrit; PDW, platelet distribution width; MPV, mean platelet volume; P-LCR, platelet large cell ratio; NEU#, absolute neutrophil count; LY#, absolute lymphocyte count; MONO#, absolute monocyte count; EOS#, absolute eosinophil count; BASO#, absolute basophil count; NEUT%, neutrophil percentage; LY%, lymphocyte percentage; MONO%, monocyte percentage; EOS%, eosinophil percentage; BASO%, basophil percentage; TT, thrombin time; PT, prothrombin time; PTR, prothrombin time ratio; INR, international normalized ratio; APTT, activated partial thromboplastin time; Fib, fibrinogen; D-dimer, D-dimer; FDP, fibrin degradation product; ALT, alanine aminotransferase; AST, aspartate aminotransferase; ALP, alkaline phosphatase; GGT, gamma-glutamyl transferase; TBIL, total bilirubin; DBIL, direct bilirubin; TP, total protein; ALB, albumin; Pre-ALB, prealbumin; TBA, total bile acid; GLU, glucose; BUN, blood urea nitrogen; Cre, creatinine; UA, uric acid; Ca, calcium; IP, inorganic phosphorus; K, potassium; Na, sodium; Cl, chlorine; TC, total cholesterol; TG, triglyceride; HDL-C, high-density lipoprotein cholesterol; LDL-C, low-density lipoprotein cholesterol; sd LDL-C, small dense low-density lipoprotein cholesterol; CK, creatine kinase; LDH, lactate dehydrogenase; tPSA, total prostate-specific antigen; fPSA, free prostate-specific antigen; f/t PSA ratio, free/total prostate-specific antigen ratio; PSAD, prostate-specific antigen density.

**Table 2:** Multivariate regression analysis of coagulation and other blood parameters with csPCa in patients undergoing prostate biopsy.

Models	Variables	β Value	Multivariate Model		
			OR	95% CI	*p* Value
1	Age	0.061	1.063	1.025–1.102	**0.001**
	BMI	0.018	1.018	0.938–1.105	0.670
	WBCs	0.026	1.027	0.889–1.185	0.719
	ALB	0.024	1.025	0.939–1.119	0.585
	Fib	−0.672	0.511	0.346–0.754	**0.001**
	PLT	−0.008	0.992	0.986–0.997	**0.005**
	PSAD	2.939	18.892	7.417–48.117	**<0.001**
2	Age	0.057	1.059	1.020–1.098	**0.002**
	BMI	0.005	1.005	0.928–1.089	0.900
	WBCs	−0.058	0.944	0.827–1.078	0.393
	ALB	0.064	1.066	0.975–1.166	0.163
	D-dimer	0.028	1.028	0.828–1.276	0.801
	PLT	−0.009	0.992	0.986–0.997	**0.003**
	PSAD	2.638	13.989	5.716–34.235	**<0.001**

Note: Multivariate regression models are shown. The dependent variable was the presence of csPCa. The bold value indicated statistical significance. Abbreviations: csPCa, clinically significant prostate cancer; BMI, body mass index; WBCs, white blood cells; ALB, albumin; Fib, fibrinogen; PLT, platelet; PSAD, prostate-specific antigen density; OR, odds ratio; 95% CI, 95% confidence interval; β, regression coefficient.

**Table 3:** Diagnostic efficacy of coagulation-derived parameters for detecting csPCa in all enrolled subjects.

Models	Variables	β Value	Multivariate Model		
			OR	95% CI	*p* Value
1	Age	0.060	1.062	1.024–1.101	0.001
	BMI	0.018	1.018	0.939–1.105	0.661
	WBCs	0.025	1.025	0.889–1.182	0.736
	ALB	0.020	1.020	0.936–1.112	0.651
	Fib-PLT Score	−0.956	0.384	0.250–0.590	<0.001
	PSAD	2.925	18.632	7.389–46.984	<0.001
2	Age	0.060	1.062	1.024–1.102	0.001
	BMI	0.015	1.016	0.935–1.103	0.716
	WBCs	0.033	1.034	0.894–1.194	0.655
	ALB	0.026	1.026	0.937–1.123	0.577
	Fib-PLT-DD Score	−0.946	0.388	0.251–0.599	<0.001
	PSAD	2.894	18.063	7.125–45.792	<0.001

Note: The *p* value was calculated to test the null hypothesis that the true AUC, 0.5. A *p*-value < 0.05 was considered statistically significant. Fib-PLT Score = 0.672 × Fib + 0.008 × PLT; Fib-PLT-DD Score = 0.672 × Fib + 0.008 × PLT − 0.028 × DD. Abbreviations: csPCa, clinically significant prostate cancer; BMI, body mass index; WBCs, white blood cell count; ALB, albumin; Fib, fibrinogen; PLT, platelet count; PSAD, prostate-specific antigen density; DD, D-dimer; OR, odds ratio; 95% CI, 95% confidence interval.

**Table 4:** Diagnostic efficacy of coagulation-related parameters for csPCa detection in all enrolled men.

Predictors	AUC	95% CI	*p* Value
Fib	0.557	0.508–0.607	0.030
PLT	0.581	0.531–0.632	0.002
D-dimer	0.447	0.394–0.501	0.047
Fib-PLT Score	0.595	0.546–0.645	<0.001
Fib-PLT-DD Score	0.602	0.553–0.652	<0.001

Note: The *p* value was calculated to test the null hypothesis that the true AUC is 0.5. A *p* value < 0.05 was considered statistically significant. Fib-PLT Score = 0.672 × Fib + 0.008 × PLT; Fib-PLT-DD Score = 0.672 × Fib + 0.008 × PLT − 0.028 × DD. Abbreviations: csPCa: clinically significant prostate cancer; AUC: area under curve; Fib: fibrinogen; PLT: platelet.

**Table 5:** Diagnostic efficacy of csPCa-DI and PSA-related parameters for csPCa detection in all enrolled men.

Predictors	AUC	95% CI	*p* Value	Cut-Off	Sensitivity	Specificity	Youden’s
csPCa-DI	0.821	0.773–0.868	<0.001	1.46	87.4%	61.8%	0.492
PSAD	0.797	0.744–0.850	<0.001	0.33	66.0%	84.3%	0.503
tPSA	0.701	0.639–0.763	<0.001	16.81	56.3%	77.0%	0.333
f/t PSA	0.727	0.680–0.774	<0.001	0.140	60.0%	80.5%	0.405

Note: The *p* value was calculated to test the null hypothesis that the true AUC is 0.5. A *p* value < 0.05 was considered statistically significant. csPCa-DI = 2.894 × PSAD + 0.060 × Age − 0.946 × Fib-PLT-DD Score. Abbreviations: csPCa-DI: clinically significant prostate cancer discrimination index; AUC: area under curve; 95% CI: 95% confidence interval; PSAD, prostate-specific antigen density; f/t PSA, free/total prostate specific antigen ratio.

**Table 6:** Diagnostic efficacy of csPCa-DI and PSA-related parameters for detecting csPCa in men with gray-zone PSA.

Predictors	AUC	95% CI	*p* Value	Cut-Off	Sensitivity	Specificity	Youden’s
csPCa-DI	0.736	0.634–0.838	<0.001	0.92	96.0%	48.0%	0.435
				1.46	68.0%	70.7%	0.387
PSAD	0.720	0.608–0.832	0.001	0.18	52.0%	83.8%	0.358
tPSA	0.465	0.349–0.581	0.586	NA	NA	NA	NA
f/t PSA	0.638	0.512–0.763	0.034	0.175	60.0%	71.7%	0.317

Note: The *p* value was calculated to test the null hypothesis that the true AUC is 0.5. A *p* value < 0.05 was considered statistically significant. Abbreviations: csPCa-DI: clinically significant prostate cancer discrimination index; AUC: area under curve; 95% CI: 95% confidence interval; PSAD, prostate-specific antigen density; f/t PSA, free/total prostate specific antigen ratio; NA, not applicable (no valid cut-off value due to non-significant diagnostic efficacy).

## Data Availability

The datasets used and/or analyzed during the current study are available from the corresponding authors on reasonable request.
